# Effect of Malunited Midshaft Clavicular Fractures on Shoulder Function

**DOI:** 10.5402/2011/507287

**Published:** 2011-07-24

**Authors:** Shachar Shapira, Zeevi Dvir, Uri Givon, Ariel Oran, Amir Herman, Moshe Pritsch (Perry)

**Affiliations:** ^1^Shoulder Surgery Unit, Department of Orthopaedic Surgery, The Chaim Sheba Medical Center, Tel Hashomer 52621, Israel; ^2^Institute of Motor Functions, Physical Therapy, The Chaim Sheba Medical Center, Tel Hashomer 52621, Israel; ^3^Motion Analysis Laboratory, The Chaim Sheba Medical Center, Tel Hashomer 52621, Israel; ^4^Department of Orthopaedic Surgery, The Chaim Sheba Medical Center, Tel Hashomer 52621, Israel; ^5^Talpiot Medical Leadership Program, The Chaim Sheba Medical Center, Tel Hashomer 52621, Israel

## Abstract

*Background and Purpose*. Displaced middle third clavicle fractures are traditionally treated non-operatively and heal with residual deformity. Few studies assessed treatment success by using patient-oriented outcome measures or objective muscle strength testing. The purpose of our study was to determine whether clavicle malunion affects functional results. *Methods*. Union was documented in 25 patients who were treated conservatively due to a displaced mid shaft clavicle fracture. Ten had significant malunion. Patients were examined at least 12 months following the fracture. Function was assessed by DASH and UCLA questionnaires. Clinical assessment included Range of Motion (ROM) measurement, manual and isokinetic muscle strength testing. Healthy shoulder served as a control. *Results*. Mean follow up time was 38 months. The mean DASH score was 9, mean UCLA score was 31.7. Range of motion was preserved—less than 6° side-to-side difference. Abduction strength reduction in the involved side amounted to 7%. No correlation was found between radiographic malunion and the functional results. *Interpretation*. Displaced healed middle clavicle fractures result in satisfactory functional results. The average deficits detected in strength and ROM were within the normal limits compared to the non-injured side. Radiographic healing position had no effect on functional outcome.

## 1. Introduction

Fractures of the clavicle account for 2.5 to 5 percent of all fractures [1, 2], with an annual incidence of 71 to 100,000 in males and 30 to 100,000 in females. These fractures involve the middle third in 69 to 82% of the cases [3–5] and are more common in children and young adults. Displaced middle third clavicle fractures are traditionally treated by closed reduction which is difficult to maintain, and those fractures usually heal with a certain amount of deformity—particularly shortening. Traditionally, the residual deformity is considered to have no significant effect on shoulder function [6]. Most of the previous studies based the success of nonoperative treatment on radiographic union which occurred in more than 95% of the cases [7, 8]. Only few studies related to patient-oriented outcome measures such as the DASH (Disabilities of the Arm, Shoulder and Hand) questionnaire or objective shoulder muscle strength testing. Some of those studies showed inferior results regarding shoulder strength and endurance [9–12] and a tendency towards poor results when the fractures healed with significant shortening or angulations [13].

Manual muscle strength testing may be normal while the more accurate objective isokinetic muscle strength testing can demonstrate reduced strength as compared to the uninvolved limb [14]. In addition, the shoulder muscles contract in different fashions—static (isometric) contractions and dynamic (concentric and eccentric) contractions. Thus, complete objective strength evaluation of the shoulder should include all types of contractions, in order to detect subtle differences in muscle strength [15].

The purpose of the present study was to compare shoulder abduction strength and range of motion in patients treated nonoperatively in order to determine if reasonable shoulder functional symmetry was achieved. Our hypothesis was that radiographic shortening, or angulations were not correlated with inferior functional results.

## 2. Materials and Methods

Based on the Shoulder Trauma Clinic records, 51 patients who had sustained a middle third clavicle fracture from the years 2003 to 2009 and who had been treated non-operatively were included in this IRB-approved study. We included patients who had reached both clinical and radiographic union and were able to complete the DASH questionnaire and to perform objective muscle strength test. We excluded 9 patients for nondisplaced fracture (2 patients), existing ipsilateral or contralateral upper-extremity limb pathology (6 patients), and open fracture (1 patient). Nine patients were soldiers on active military service in distant units who were unavailable for our followup (all of them serving in fighting units involving strenuous physical activities). Eight patients refused to participate in our study. Eventually, twenty-five patients who met all inclusion criteria completed the study—20 males and 5 females. The average age was 33.7 years (ranging 18 to 48). Fractures were caused by a bicycle accident in 6 patients, a motorcycle accident in 8 patients, a motor vehicle accident in 7 patients, and a fall in 4 patients.

Ten patients (40%) were professional or semiprofessional athletes. Patients were examined at least 12 months following the fracture. Radiographic evaluation was conducted using PACS (Philips, The Netherlands) software. We used standard 2 radiographs for each patient—a shoulder anteroposterior view and shoulder Anteroposterior with cephalic 20 degrees tilt. We measured the angulations, shortening and displacement on both final follow-up radiographs, and the worst measurement was recorded. Fracture malunion was defined if one of the following criteria [7, 10] was present: angulation of more than 15 degrees, shortening, or displacement of more than 1.5 cm. Functional assessment was performed using the DASH and UCLA questionnaires. The DASH questionnaire was used for subjective assessment: a score of 0 was associated with no disability while a score of 100 was considered maximal disability. The score addresses pain, function, and social problems. The UCLA questionnaire was used for objective functional assessment; the maximum score is 35 points; grade <27 is considered as fair/poor (unsatisfactory result) and grade >27 is considered excellent/good (satisfactory result). Clinical assessment included range of motion (ROM) measurement using a goniometer—abduction, forward flexion, elevation, adduction, and internal and external rotation were measured on both sides. The manual muscle testing and all the other measurements were performed by the same physician.

Isokinetic muscle strength testing was performed using a CYBEX Norm dynamometer. A standard shoulder abduction and adduction muscle strength protocol was applied at two different velocities: 60 and 120°/s. Measurements were taken while the patient seated in fixed position and the arm stabilized with straps. The lever arm height was adjusted according to the shoulder center of rotation, and the range of motion was limited between 60 to 150 degrees of abduction. We evaluated the healthy shoulder that served as a control first, followed by the healed one. Each patient performed a warm-up session followed by the test session that contained five repetitions at each velocity. We then evaluated the peak muscle strength. Difference between shoulders for both range of motion and maximum strength was calculated as percentage from the uninjured shoulder.

Statistical analysis was done using SPSS (c) 16.0 (Chicago, Ill, USA) by an experienced biostatistician (A. H.). Categorical data is presented as count (percent). Continuous data is presented as mean (range  =  min − max, SD  =  standard deviation). Comparisons of continuous variables between patients with or without fracture malunion were done using the chi-square test. Comparisons of continuous variables between patients with or without fracture malunion were done using the Wilcoxon-Mann-Whitney rank sum test. All *P* values reported are two sided. *P* value below or equal 0.05 was considered statistically significant.

## 3. Results

The mean follow-up time was 38 months following the fracture—ranging from 12 to 90 months. Nine patients were examined 12 to 23 months following the fracture. The dominant limb was involved in 12 patients (48%). Radiographic and clinical healing was achieved after a mean time of 3.56 months (range: 1.5–10 m, SD = 1.9). In 18 of 25 (72%), healing lasted less than 3 months. The mean angulation was 5.5° (range: 0 to 23°, SD = 9.11), mean shortening 0.8 cm (range: 0.1 to 1.6 cm, SD = 0.53), and mean displacement was 1.05 cm (range: 0.2 to 2.1 cm, SD = 0.59). Ten patients out of 25 (40%) had fracture malunion. There were no statistically significant differences in demographic characteristics between patients with or without fracture malunion (see Table 1).

### 3.1. Patient-Oriented Outcome Measures

**DASH Score**

The mean DASH score was 9 (range: 0–43.3, SD = 11.59). Twenty-three out of 25 patients (88%) had a DASH score lower than 23 points. No statistically significant difference in DASH score was found between patients without fracture malunion (mean DASH score 10.6 ranging 0–43) and patients with fracture malunion (mean DASH score 6.59 ranging 0–34).

**UCLA Score**

The mean UCLA score was 31.7 (range: 22–35, SD = 3.55). Twenty-three out of 25 patients (88%) had UCLA score >27 representing satisfactory result. The 2 patients who scored 22 and 26 points had a radiographic shortening of 1.3 cm without angulation. No statistically significant difference in the UCLA score was found between patients without fracture malunion (mean UCLA score 31.1, range 22–35) and patients with fracture malunion (mean UCLA score 32.6, range 26–35).

**Range of Motion**

The ROM was well preserved above the functional demands. We found an average 6° side-to-side difference in forward flexion (173.6° on the injured side and 179.6° on the normal side) and abduction (174° on the injured side and 180° on the normal side). Six patients showed side-to-side difference in forward flexion and abduction ranging 10–50° difference (1 patient showed better ROM on the injured side). External rotation was averagely decreased by 4° (83.6° on the involved side and 87.6° on the normal side), and a side-to-side difference was found in 4 patients (range: 10–30°). Internal rotation was decreased on the involved side in 3 patients—1 had slight restriction reaching D8 vertebra on the involved side and 2 reaching D12 vertebra versus D7 on the normal side. No statistically significant difference was found in shoulder ROM differences between patients with or without fracture malunion. Mean abduction ROM side-to-side difference was 3% in both groups (range 0–28%). Mean forward flexion ROM side-to-side difference was 3% (range 6%–28%) in the good union group and 4% (range 0–33%) in the malunion group (*P*  value = 0.807). Mean External rotation ROM side-to-side difference was 3% (range 0–33%) in the good union group and 7% (range 0–44%) in the malunion group, with *P* value = 0.765).

**Strength**

Manual strength testing: 23 of 25 patients (88%) had bilateral 5/5 gross muscle strength in abduction, forward flexion, and external and internal rotation. Two patients had 4/5 abduction and elevation strength on the involved side compared to 5/5 on the normal side. No correlation was found between strength decrease and the radiographic appearance.

Isokinetic strength: abduction strength reduction in the involved side amounted to 7.3% at 60°/s and 3.5% at 120°/sec, both insignificant. Five patients (20%) had a significant interextremity difference of more than 20% of peak abduction strength at 60°/s, 4 had shown increased strength of the healthy shoulder and 1 of the injured shoulder. Nine patients had a significant interextremity difference of peak abduction strength at 120°/s; six had shown increased strength on the healthy side and 3 on the injured side. No statistically significant difference was found between the radiographic malunion and strength difference between the shoulders (Table 2).

**Return to Sports**

Ninety percent (9/10) of the professional athletes among the cohort returned to sporting activities. No statistically significant difference was found between athletes with or without malunion and the return to sports activities (Table 1).

## 4. Discussion

Nonoperative treatment is well accepted in patients with middle third clavicle fractures. A relatively high malunion rate is usually associated with satisfactory results regarding pain. Nordqvist rated 40 of 53 patients with malunited fractures of the clavicle as good [16]. Midterm and long-term follow-up revealed inconclusive results regarding pain when significant displacement or shortening of the fracture had occurred; Eskola et al. had shown inferior results [17] while Oroko et al. [18] and Nordqvist et al. [19] had shown good results in most patients. Some of those studies were criticized because their end result was pain and not functional outcome. Recent studies [10, 11, 13] demonstrated a tendency towards functional impairment associated with the radiographic displacement or shortening, implying that surgical treatment should be considered. The mechanical basis for that assumption was that shortening or angulation can decrease the moment arm of the shoulder abductors resulting in decreased strength and range of motion. Some of those series [11] had a small percentage of follow-up (30 of 107 patients at one study). In our series, 25 of 51 patients were available for complete follow up, and 9 more were soldiers on active military duty who were unavailable for complete examination.

We found satisfactory results of the DASH and UCLA scores in most patients, as well as preserved ROM with minor and clinically insignificant loss of elevation and abduction compared to the uninvolved shoulder. In all of our patients, the motion was well within the functional range. There was no correlation or association of the functional results to the radiographic malunion.

The main variable tested in our study was objective strength at different angular velocities. We postulated that it can be more sensitive than the conventional subjective muscle strength evaluation which was normal in 23 of 25 patients (88%). Normally, a limb-to-limb difference of up to 10 % is considered normal, of 10 to 20% is considered probably significant, and above 20% is considered significant [14]. The difference is not necessarily in favor of the dominant limb. The average loss of strength was below 10% in the slow and fast angular velocities, matching the patients ability to perform different overhead swing activities at different resistance (overhead sports like volleyball, basketball, and cliffhanging as well as weight lifting). There was a slight tendency toward a higher reduction of strength at the lower angular velocity (7.3% at 60°/s versus 3.5% at 120°/s). Strength restoration can be explained by the intensive physiotherapy performed after healing of the fracture: 1 of 5 patients had superior result in the injured shoulder at slow velocity and 3 of 9 at high velocity, even when demonstrating a significant displacement or shortening on the final follow-up radiographs.

Further studies, as conducted at other institutes or at our institute evaluating the same outcome parameters following operative treatment (usually performed in patients with clavicle fractures that are initially largely displaced), are needed in order to find whether better functional results are achieved.

## 5. Conclusions

Our results indicate that displaced or angulated healed middle clavicle fractures result in satisfactory subjective and objective functional results regarding pain, range of motion, and strength at different angular velocities. The residual average deficits detected in strength and ROM were within the normal limits when compared to the noninjured side. The radiographic healing position had no effect on shoulder functional outcome. Further studies are needed to assess whether surgical treatment leads to superior results.

## Figures and Tables

**Table 1:** Demographic data.

	Good union (*N* = 15)	Malunion (*N* = 10)	*P* value
Gender			
Male	10 (66.7%)	10 (100%)	
Female	5 (33.3%)	0 (0%)	0.041
Age	33 (range = 18–48, SD = 9.72)	34.6 (range = 20–44, SD = 7.39)	0.643
Body mass index	24.2 (range = 19.5–29.5, SD = 3.6)	25.05 (range = 22.2–32.0, SD = 3.26)	0.643
Follow-up time (months)	39 (range = 13–90, SD = 25)	36.4 (range = 12–80, SD = 19.3)	1
Time to union (months)	3.87 (range = 2–10, SD=2.15)	3.10 (range = 2–6, SD = 1.43)	0.322
Dominant hand			
Right	14 (93,3%)		
Left	1 (6.7%)	10 (100%)	0.405
Dominant side broken	7 (46.7%)	5 (50%)	0.870
Mechanism of injury:			
MVA	7 (46.7%)	0 (0%)	
Bicycle accident	3 (20%)	3 (30%)	
Motorcycle accident	3 (20%)	5 (50%)	
Fall	2 (13.3%)	2 (20%)	0.08
Prior sport activity	6 (40%)	4 (40%)	1
Return to sport of prior sport-active patients (*N* = 10)	6 (100%)	3 (75%)	0.197

**Table 2:** Outcome measurements.

	Good union (*N* = 15)	Malunion (*N* = 10)	*P* value
DASH	10.61 (range = 0–43, SD = 12)	6.59 (range = 0–34, SD = 10.7)	0.23
UCLA	31.1 (range = 22–35, SD = 3.7)	32.6 (range = 26–35, SD = 3.27)	0.285
Abduction maximum, strength at 60°/s; difference	−5.27% (range = −48%–25%, SD = 18.5)	−10.4% (range = −52%–19%, SD=23.9)	0.765
Abduction maximum, strength at 120°/s; difference	−4.93% (range = −58%–33%, SD = 21.71)	−1.3% (range = −42%–30%, SD = 26.71)	0.605
Adduction maximum, strength at 60°/s; difference	2.67% (range = −39%–54%, SD = 27.42)	−3.2% (range = −45%–18%, SD = 21.32)	0.605
Adduction maximum, strength 120°/s; difference	−0.93% (range = −80%–45%, SD = 35.66)	−16.1% (range = 48%–9%, SD=21.26)	0.103
Forward flexion difference	3% (range = −6%–28%, SD = 8)	4% (range = 0%–33%, SD = 10)	0.807
Abduction difference	3% (range = 0%–28%, SD = 8)	3% (range = 0%–28%, SD = 9)	0.807
Extension difference	1% (range = 0%–22%, SD = 6)	0% (range = 0%–0%, SD = 0)	0.807
External rotation difference	3% (range= 0%–33%, SD = 9)	7% (range = 0%–44%, SD = 15)	0.765

*Ta* *bl* *e*  2* presents patient measured outcome.* Abduction and adduction strengths were measured at angular speed of 60 °/s and 120 °/s. Peak strength was used. The difference between injured and healthy shoulder is given in percentage from the uninjured shoulder.
